# Unhealthy Lifestyle, Genetics and Risk of Cardiovascular Disease and Mortality in 76,958 Individuals from the UK Biobank Cohort Study

**DOI:** 10.3390/nu13124283

**Published:** 2021-11-27

**Authors:** Katherine M. Livingstone, Gavin Abbott, Joey Ward, Steven J. Bowe

**Affiliations:** 1 Institute for Physical Activity and Nutrition Research, School of Exercise and Nutrition Sciences, Deakin University, Geelong, VIC 3220, Australia; gavin.abbott@deakin.edu.au; 2 Institute of Health and Wellbeing, University of Glasgow, Glasgow G12 8RZ, UK; Joey.Ward@glasgow.ac.uk; 3 Deakin Biostatistics Unit, Deakin University, Geelong, VIC 3220, Australia; s.bowe@deakin.edu.au

**Keywords:** healthy lifestyle, genetic risk, cardiovascular disease, mortality, myocardial infarction, stroke

## Abstract

To examine associations of unhealthy lifestyle and genetics with risk of all-cause mortality, cardiovascular disease (CVD) mortality, myocardial infarction (MI) and stroke. We used data on 76,958 adults from the UK Biobank prospective cohort study. Favourable lifestyle included no overweight/obesity, not smoking, physical activity, not sedentary, healthy diet and adequate sleep. A Polygenic Risk Score (PRS) was derived using 300 CVD-related single nucleotide polymorphisms. Cox proportional hazard ratios (HR) were used to model effects of lifestyle and PRS on risk of CVD and all-cause mortality, stroke and MI. New CVD (*n* = 364) and all-cause (*n* = 2408) deaths, and stroke (*n* = 748) and MI (*n* = 1140) events were observed during a 7.8 year mean follow-up. An unfavourable lifestyle (0–1 healthy behaviours) was associated with higher risk of all-cause mortality (HR: 2.06; 95% CI: 1.73, 2.45), CVD mortality (HR: 2.48; 95% CI: 1.64, 3.76), MI (HR: 2.12; 95% CI: 1.65, 2.72) and stroke (HR:1.74; 95% CI: 1.25, 2.43) compared to a favourable lifestyle (≥4 healthy behaviours). PRS was associated with MI (HR: 1.35; 95% CI: 1.27, 1.43). There was evidence of a lifestyle-genetics interaction for stroke (*p* = 0.017). Unfavourable lifestyle behaviours predicted higher risk of all-cause mortality, CVD mortality, MI and stroke, independent of genetic risk.

## 1. Introduction

Cardiovascular disease (CVD) is a leading cause of morbidity and mortality worldwide [1]. Risk of CVD is the result of a combination of risk factors, including non-modifiable genetic pre-disposition and a range of modifiable lifestyle behaviours, such as smoking, sleep duration, physical activity and diet [2]. Understanding of the effect of both lifestyle behaviours and genetics on CVD risk is thus important for reducing the global burden of CVD.

Limiting unhealthy lifestyle behaviours has been associated with lower risk of all-cause and CVD mortality [3,4,5]. The evidence for established modifiable risk factors, such as being physically active, not smoking, and maintaining a healthy body mass index (BMI) and a healthy diet, is strong [5,6]. However, increasing research suggests more time spent sitting during work and leisure time is also an important predictor of all-cause mortality [7], as well as too much or too little time spent sleeping [8]. As lifestyle behaviours tend to cluster and have synergistic effects on diseases [5,9], it is critical to determine the combined effects of lifestyle risk factors on health outcomes. Research from large UK, US and Korean cohorts have shown an additive benefit of maintaining multiple healthy lifestyle behaviours for reducing risk of CVD and all-cause mortality [9,10,11,12]. However, a paucity of studies has included emerging behavioural risk factors of sedentary time and sleep duration when deriving health behaviour scores [11,13,14]. Moreover, most studies have used single foods as a dietary indicator, which is not reflective of how foods are consumed together as part of an overall dietary pattern [15]. Thus, with poor diet now the leading cause of death globally [6], there is a need to use an internationally relevant indicator of overall diet quality, such as the WHO Healthy Diet Indicator (HDI) [16]. Estimating an overall lifestyle score based on existing and emerging modifiable risk factors will inform lifestyle-based guidelines for the primary prevention of CVD.

Understanding the role of unhealthy lifestyle behaviours and genetics on CVD risk is critical for advancing the design of tailored dietary interventions [9,17,18,19]. Polygenic risk scores (PRS)combine numerous Single Nucleotide Polymorphisms (SNPs) and have been shown to adequately reflect risk for multifactorial conditions, such as CVD [9,20]. Previous applications of CVD and CVD-related PRSs in the UK Biobank indicate that an unhealthy lifestyle and a PRS are independent predictors of incident hypertension, stroke and CVD, with limited evidence of interactions [9,10].

To our knowledge no studies have examined interactions between unhealthy lifestyle, a PRS and risk of both CVD and all-cause mortality. Moreover, the role of a lifestyle score based on established and emerging risk factors is unclear. Longitudinal research is needed to determine whether these lifestyle behaviours are risk factors for CVD and all-cause mortality independent of genetic risk. Further, investigation of modifiable risk factors for incidence of CVD subtypes will inform secondary prevention of CVD and CVD-mortality. Therefore, this study aimed to examine the prospective association between an unhealthy lifestyle score and a PRS and risk of all-cause mortality, CVD mortality, stroke and myocardial infarction (MI).

## 2. Materials and Methods

### 2.1. Study Design and Participants

The UK Biobank is a population-based prospective cohort study of 502,536 adults aged 40 to 69 living in the United Kingdom (UK) with data on determinants of disease [21]. Individuals were identified from patient registers of the National Health Service and were invited to participate between 2006 and 2011 by attending one of 22 assessment centres across the UK. Participants self-reported information via a touchscreen questionnaire at each centre to record information on socio-demographic characteristics, lifestyle risk factors and general health. An online 24-h dietary assessment tool, the Oxford WebQ, was used to record dietary intake data [22]. Anthropometric measurements were taken. Health records and death registries were linked to participant data. The UK Biobank received ethical approval from the Research Ethics Committee (Reference 11/NW/0382). All participants provided electronic signed consent. Participants were excluded from the present analysis if they (i) had a history of CVD before entering the study, had a CVD event during dietary exposure period, were pregnant, had implausible physical activity data, (ii) had <2 timepoints of dietary data from February 2011 to June 2012, (iii) did not identify as White British, (iv) had data missing for the exposure, outcomes, or covariates/moderators. The STROBE checklist for reporting of cohort studies was used (Table S1).

### 2.2. Lifestyle Behaviours

We derived an unhealthy lifestyle score based on six established and emerging risk factors for mortality and CVD [12,14]. Established risk factors included diet quality, physical activity, smoking, and BMI; emerging risk factors included sleep duration and sedentary time. Based on previously published health behaviour scores [9,10,12], participants were allocated 1 point for each of the six favourable lifestyle behaviours and we classified participants into one of three categories: unfavourable lifestyle (0 or 1 health behaviours); intermediate lifestyle (2 or 3 health behaviours); favourable lifestyle (4 or more health behaviours). For sensitivity analyses we treated the lifestyle score as a continuous variable.

### 2.3. Diet Quality

Diet quality was estimated from dietary data collected using the OxfordWebQ. The Oxford WebQ was used to record the frequency of intake of 32 beverages and 206 foods during the past 24-h [22,23,24], and has been validated against total energy expenditure, biomarkers and an interviewer-administered multiple-pass 24-h dietary recall [24]. Dietary intakes were estimated from the frequency of intake of each food or beverage, standard portion sizes and the composition of each item [25,26]. From April 2009 to September 2010, participants completed the 24-h dietary assessment using the touchscreen at the assessment centre. Between February 2011 to June 2012, four repeat online assessments were collected. We calculated mean baseline dietary intake for participants who had ≥2 valid measurements using the four online cycles (February 2011–June 2012) as 16 months was considered a more credible timeframe.

Information on dietary intake was used to calculate the HDI. This index was selected as it represents an internationally relevant diet quality methodology that has been applied internationally to assess diet-disease associations which has been previously used in the UK Biobank [16,18,27,28,29,30,31]. The HDI is a food- and nutrient-based index that reflects consumption of foods recommended by the World Health Organisation for a healthy diet [32]. The 12-point score designed by Maynard et al. [30] was adapted by removing cholesterol intake, which was not part of the 2020 World Health Organisation healthy diet fact sheet [32]. The resulting 11-item score was comprised of the following items: poly-unsaturated fat; saturated fat; total carbohydrates; dietary fibre; protein; fruits and vegetables; fish; red meat and meat products; pulses and nuts; total non-milk extrinsic sugars; and calcium. Information on non-milk extrinsic sugars intake was not available, so we used intake of total sugars instead (Table S2). Cut offs were used to assign a score of 1or 0. The total HDI score range was from 0 to 11, with a higher score reflecting a higher diet quality (Table S3). Based on previous definitions [27], a favourable diet quality was classified as ≥median HDI score (median was 2.0).

### 2.4. Other Lifestyle Behaviours

Smoking habits (never, previous and current smoker) were collected; favourable smoking habits were classified as never smoked or previous smoker. BMI was derived as weight (kg)/height (m)^2^. Favourable BMI was estimated by creating a binary variable to reflect overweight/obesity based on standard World Health Organisation cut offs [33]. Physical activity was determined from a modified version of the International Physical Activity Questionnaire [34]. Information on walking, moderate, and vigorous physical activity undertaken over the last 7 days was used to estimate Metabolic Equivalents (METs), where one MET was defined as the energy cost of sitting quietly and is equivalent to a caloric consumption of 1 kcal/kg/hour. We categorised participants as physically active based on meeting physical activity guidelines of 150 min per week if their METs were ≥600 MET-min/week [34]. Time spent watching TV and using the computer were used to estimate favourable sedentary time (hours/day), classified as ≤7 h/day based on previous use of these variables in the UK Biobank [35,36]. We classified favourable sleep duration based on ≥7 and ≤9 h sleep/night [37].

### 2.5. Polygenic Risk Score

Entered genetic data from the UK Biobank (downloaded 11 November 2019) were used. In addition to exclusion criteria listed previously, we excluded participants who were missing >10% of their genetic data and participants were identified as being heterozygosity outliers by UK Biobank. Further, for every pair of individuals who were second cousins or closer (i.e., participants with a kinship coefficient greater than 0.042) one was excluded at random. We estimated a PRS for CVD based on 300 SNPs with established associations with coronary artery disease [38] PLINK, an open-source platform for genomic research, was used to derive the PRS. Firstly, the sum of the number of risk alleles present at each locus was derived and weighted by the log of the odds for that locus [20], estimated from the list of 300 SNPs using the PLINK “–score” command—with no-mean-imputation flag. PRSs were standardised and treated as a continuous variable in all modelling.

### 2.6. Cardiovascular Events and Mortality

Mortality status and causes of death were established by data linkage with the UK National Death Index (NDI). The accuracy of the NDI for classifying CVD deaths has been established previously in Australia [39]. CVD mortality was estimated from death certificate 2006 International Classification of Diseases 10th revision (ICD-10) codes I05-I89. CVD events were identified between enrolment and in the latest available inpatient hospital data. Incident stroke (ischaemic, intracerebral haemorrhage, and subarachnoid haemorrhage) and MI (ST-Elevation Myocardial Infarction and Non-ST-Elevation Myocardial Infarction) were available from algorithms provided by the UK Biobank [40,41]. Algorithms were derived to identify incident cases using hospital and death register data, as detailed elsewhere [40,41]. A censoring date of 4 March 2020 was used due to a spike in deaths from 5 March onwards, which corresponds to increasing deaths due to COVID-19 recorded in the UK.

### 2.7. Demographic and Health Information

At recruitment, interview-administered questionnaires were used to collect information on demographic characteristics and medical history. At recruitment, age and sex were self-reported, with no adjustments performed for discrepancies between genetic sex and self-reported sex. The Townsend deprivation index was estimated at baseline, representing an aggregate measure of deprivation based on unemployment, non-home ownership, non-car ownership, and household overcrowding [42], where a score was assigned corresponding to the postcode of each participants’ home dwelling; a negative value represented high socioeconomic status. We categorised the deprivation index into quintiles. Information was collected on use of medication (anti-hypertensive, lipid-lowering or exogenous hormones or diabetes; yes, no) and doctor diagnosis of any type of diabetes or a CVD event (yes, no). A binary variable was created representing family history of CVD and CVD-related diseases (yes/no).

### 2.8. Statistical Analysis

We used complete case analysis. Missingness was examined by comparing demographic characteristics of the excluded sample with the analytic sample. Descriptive analyses included number (%) for categorical variables and mean (SD) for continuous variables.

Multivariable Cox proportional hazard regression models were used to approximate hazard ratios (HR) and 95% Confidence Intervals (CI) of all-cause mortality, CVD mortality and risk of CVD subtypes (MI and stroke) according to an unhealthy lifestyle score (categorical independent variable). We treated CVD events and mortality as outcome/dependent variables. The time scale used was age (years). The duration of follow up was the time between the last day of dietary data and incident event, MI, stroke or death or the censoring date (4 March 2020). For participants who had more than one events during the study period, the first event date was used. Cox regression analyses were adjusted for age (timescale), sex and deprivation (categorical). Unhealthy lifestyle by sex interactions were examined by including an interaction term in the model. In accordance with guidelines for reporting of for sex differences in CVD research [43], analyses were presented stratified by sex. The Cox proportional hazards models also incorporated PRS as an independent variable and included a covariate to represent the first 8 principal components of ancestry and genotyping batch [9]. We added an interaction term to the models to test for interaction between lifestyle score and PRS. Where there was evidence effects of lifestyle score were moderated by PRS (*p* < 0.05 for interaction term), interaction effects were explored by conducting post-hoc estimation of the effects of unhealthy lifestyle on events at “low” (−1 SD) and ‘high’ (+1 SD) PRS score. To investigate reverse causation, sensitivity analyses excluded deaths and incident cases within the first 2 years of follow up. Cox proportional hazard regression models were also used in sensitivity analysis to estimate risk of all outcomes according to lifestyle score treated as a continuous independent variable (range 0–6). Data were analysed using Stata (version 16.0; StataCorp., College Station, TX, USA).

## 3. Results

Of the 502,536 participants recruited at baseline into the UK Biobank, *n* = 425,529 were excluded for having unusable genetic data (*n* = 1459), not being white British (*n* = 92,907), being ineligible (*n* = 23,215) or missing data (*n* = 307,997; Figure S1). Compared to the included sample, excluded participants were comparable in age and sex, with slightly higher BMI and smoking and deprivation rates (Table S3). A total of 76 958 participants were included in this analysis (Table 1). At recruitment, 55% were female and mean age was 56.2 (SD 7.8) years. Most participants were experiencing low to mid deprivation (67%). Ninety-four percent were non-smokers, 39% did not have overweight/obesity, 30% were physically active, 54% had a healthy diet, 95% were not sedentary and 79% had optimal sleep. 56% of participants had 4 or more favourable lifestyle behaviours, while 41% had two or three, and 3% had either none or one favourable lifestyle behaviour (Table 1).

Over a mean follow-up of 7.8 years (603,638 person-year), there were 364 deaths due to CVD and 2408 all-cause deaths. Over a mean follow-up of 7.8 years (601,475 person-years), there were 748 new stroke and 1140 new MI events. Of these, the majority of CVD (72%) and all-cause (59%) deaths and stroke (60%) and MI (72%) events were in males.

### 3.1. Unhealthy Lifestyle and Risk of All-Cause Mortality

An unfavourable lifestyle (0 or 1 favourable behaviours) was associated with higher risk of all-cause mortality (HR: 2.06; 95% CI: 1.73 to 2.45) compared to a favourable lifestyle (4 or more favourable behaviours; Table 2). There was no evidence (all *p*-values > 0.05) of sex by healthy lifestyle score interactions. Associations were similar in men and females. There was limited evidence of an association between PRS and all-cause mortality and a PRS by healthy lifestyle score interaction (*p*-interaction = 0.34). Effect sizes were consistent when deaths within the first 2 years of follow up were excluded (data not shown), and results were congruent when the healthy lifestyle score was treated as a continuous variable (Table S4).

### 3.2. Unhealthy Lifestyle and Risk of CVD Mortality

An unfavourable lifestyle (0 or 1 favourable behaviours) was associated with higher risk of CVD mortality (HR: 2.48; 95% CI: 1.64 to 3.76) compared to a favourable lifestyle (4 or more favourable behaviours; Table 2). There was no evidence of sex by lifestyle score interactions. Associations were comparable in men, while there was limited evidence of an association between lifestyle score and CVD mortality in females. There was some evidence of an association between PRS and CVD mortality (HR: 1.11, 95% CI: 1.00, 1.23). There was no evidence of interaction between lifestyle score and PRS for CVD mortality (*p*-interaction = 0.39). Effect sizes were consistent when deaths within the first 2 years of follow up were excluded (data not shown) and results were congruent when the healthy lifestyle score was treated as a continuous variable (Table S4).

### 3.3. Unhealthy Lifestyle and Risk of Non-Fatal CVD Events

An unfavourable lifestyle (0 or 1 favourable behaviours) was associated with higher risk of MI (HR: 2.12; 95% CI: 1.65 to 2.72) and stroke (HR: 1.74; 95% CI: 1.25 to 2.43) compared to a favourable lifestyle (4 or more favourable behaviours; Table 2). There was evidence of sex by lifestyle score interactions for stroke only (p-interaction=0.020). There was strong evidence of an association between PRS and MI (HR: 1.35; 95% CI: 1.27, 1.43). There was evidence of interaction between lifestyle score and PRS for stroke only (*p*-interaction = 0.017). There was no evidence of an effect of lifestyle score on stroke for participants with low PRS (HR: 1.04, 95% CI: 0.87 to 1.25, *p* = 0.64), however there was strong evidence of an association between higher unhealthy lifestyle score and higher risk of stroke events for those with high PRS (HR: 1.41, 95% CI: 1.19 to 1.67, *p* < 0.001). Effect sizes were comparable when incident MI and stroke cases within the first 2 years of follow up were excluded (data not shown) and results were congruent when the healthy lifestyle score was treated as a continuous variable (Table S4).

## 4. Discussion

This prospective population-based cohort study of more than 76,000 adults aimed to examine the association of an unhealthy lifestyle score based on smoking status, BMI, diet quality, physical activity, sleep duration and sedentary time, and a genetic risk score with all-cause and CVD mortality and non-fatal CVD events up to 8 years later. Our main findings were that a greater number of unfavourable lifestyle behaviours was associated with substantially higher risk of mortality and stroke and MI, regardless of genetic CVD risk. We observed that higher genetic risk of CVD was associated with MI only. The presence of an interaction suggests an unhealthy lifestyle may exacerbate higher risk of stroke in individuals with high genetic risk of CVD. Nevertheless, findings from this study reinforce the benefit of following a healthy lifestyle independent of genetic risk.

Our outcomes are consistent with the broader literature reporting lower risk of all-cause and CVD mortality and non-fatal CVD events with healthier lifestyle behaviours [5,9,10,12]. In a population-based cohort study using data from 44,462 US adults and 399,537 UK adults, a healthy lifestyle score based on no heavy alcohol consumption, never smoking, being more physical active, and having higher dietary quality was associated with lower risk of all-cause and CVD mortality up to 11 years later [5]. Similarly, across four studies involving 55,685 adults, a favourable lifestyle (three of the following behaviours: no current smoking, no obesity, regular physical activity, or a healthy diet) was associated with susceptibility to coronary artery disease up to 21 years later [12]. To our knowledge, no studies have examined risk of all-cause mortality, CVD mortality or non-fatal CVD events using a lifestyle behaviour score that includes all six behaviours used in the present study. Nonetheless, our results are consistent with health behaviour scores that have used either sedentary time [14] or sleep [11]. Further research is needed to replicate our unhealthy lifestyle score in independent populations.

This study confirms previous research showing limited evidence for interactions between genetics and lifestyle, despite genetic risk being associated with higher risk of non-fatal CVD events [9,12,18]. In a study of 339 003 adults, there was higher risk of coronary artery disease and stroke in individuals with higher genetic CVD risk and least favourable lifestyle behaviours (based on smoking, BMI, physical activity and diet) compared to those with lower risk and more favourable behaviours, however, no statistically significant interactions were observed [10]. Similarity, other studies using lifestyle scores that assessed risk of CVD mortality [44] or non-fatal events [9,12] have showed limited evidence of interactions. Despite a lack of consistent evidence for interactions, these studies still report up to 5-fold higher risk of coronary artery disease in participants with a poor lifestyle and highest PRS [10], which is consistent with the high risk of stroke observed in participants in this study with unhealthy lifestyle and high PRS. Nonetheless, the inconsistent evidence suggests that maintaining a healthy lifestyle remains important for all participants, regardless of genetic risk. With the growing traction of personalised diet and health advice [45], whether population groups would benefit from different lifestyle advice based on their genetic pre-disposition to CVD remains unclear. As the majority of large-scale research to date on lifestyle-gene interactions has been conducted in Caucasian populations [9,10], further high-quality research in more ethnically diverse populations is needed to determine the applicability of personalised lifestyle interventions based on genetic information. Moreover, the potential to successfully change and maintain lifestyle behaviours is likely to be dependent on the behaviour change strategies used, and whether support is personalised based on more than just genetic information [19]. Further, health professionals need to be provided with the necessary training to increase genetic literacy and their ability to effectively communicate genetic advice [45].

### 4.1. Implications of This Research

These findings have implications for lifestyle recommendations provided in clinical practice and for the design of guidelines for the primary prevention of CVD. Our results indicate that individuals would benefit from interventions and policies that aim to improve risk factors that are commonly targeted, such as a diet and physical activity, as well as emerging risk factors of sedentary time and sleep duration. As multi-component interventions are commonly used in the primary and secondary prevention of CVD [46], these findings support the benefit of designing interventions and policies that help address multiple risk factors. Since we observed some evidence of an interaction between unhealthy lifestyle and genetics on stroke, further research should explore whether genetic pre-disposition should be incorporated into clinical recommendations for CVD prevention.

### 4.2. Strengths and Limitations

The primary strengths of this study were the large sample size and creation of a genetic risk score The PRS used in this study was based on 300 SNPs and has been used previously to detect predispositions to CVD and mortality. The dietary questionnaire used was validated and enabled us to derive an overall diet quality index based on WHO dietary recommendations. We acknowledge a number of limitations. The dietary assessment tool is a short-term measure of intake, however, the use of up to four online cycles in the present study provided a longer-term estimate of intake. Our analysis is expected to be impacted by self-selection bias in the participants who completed the dietary assessment. Our sample included only participants who identified as white British, and thus cannot be generalised to a non-white population. Although our measure of sedentary time has been used in previous research [35,36], future research should derive a measure of sedentary time and bouts from direct measures, such as accelerometers. Lastly, whilst we adjusted analyses for relevant confounders based on the literature, we cannot discount the potential for unmeasured or residual confounding.

### 4.3. Conclusions

Findings from this prospective population-based cohort study suggest an unhealthy lifestyle, based on smoking, having overweight or obesity, having lower diet quality, sub-optimal sleep duration, being less physically active, and higher sedentary time was associated with higher risk of all-cause and CVD mortality and non-fatal CVD events, regardless of genetic CVD risk. Regardless of genetic predisposition to CVD, our results suggests that individuals would benefit from improving established risk factors, such as a diet and physical activity, as well as emerging risk factors of sedentary time and sleep duration. As we observed some evidence of an interaction between unhealthy lifestyle and genetics on stroke, further research should explore whether genetic pre-disposition should be incorporated into clinical recommendations for CVD prevention. Future research should also aim to replicate these findings in more racially diverse populations.

## Figures and Tables

**Table 1:** Characteristics of participants at baseline in the UK Biobank.

Characteristic	Overall *N* (%)	Males *N* (%)	Females *N* (%)
*n*	76,958	34,968 (45.4)	41,990 (54.6)
Age at recruitment (years), Mean ± SD	56.2 ± 7.8	57.0 ± 7.8	55.6 ± 7.7
Townsend Deprivation Index ^1^			
Least deprived	18,115 (23.5)	8605 (24.6)	9510 (22.7)
2nd least deprived	17,222 (22.4)	7910 (22.6)	9312 (22.2)
Medium deprivation	16,062 (20.9)	7156 (20.5)	8906 (21.2)
2nd most deprived	14,891 (19.4)	6547 (18.7)	8344 (19.9)
Most deprived	10,668 (13.9)	4750 (13.6)	5918 (14.1)
Body Mass Index (kg/m^2^), Mean ± SD	26.5 ± 4.4	27.1 ± 3.9	26.0 ± 4.7
Waist circumference (cm), Mean ± SD	88.1 ± 13.0	95.2 ± 10.8	82.3 ± 11.6
Total PA (MET min), Mean ± SD	2477 ± 2326	2542 ± 2439	2423 ± 2227
Medication use ^2^	16,562 (21.5)	9707 (27.8)	6855 (16.3)
Family history of CVD	57,182 (74.3)	25,068 (71.7)	32,114 (76.5)
Favourable lifestyle behaviours ^3^			
Non-smoker	71,995 (93.6)	32,305 (92.4)	39,690 (94.5)
No overweight/obesity	30,173 (39.2)	10,543 (30.2)	19,630 (46.8)
Physically active	23,131 (30.1)	10,589 (30.3)	12,542 (29.9)
Healthy diet	41,877 (54.4)	18,495 (52.9)	23,382 (55.7)
Not sedentary	73,305 (95.3)	32,821 (93.9)	40,484 (96.4)
Optimal sleep	60,545 (78.7)	27,364 (78.3)	33,181 (79.0)
Lifestyle score			
4 or more favourable lifestyle behaviours	43,118 (56.0)	17,516 (50.1)	25,602 (61.0)
2 or 3 favourable lifestyle behaviours	31,363 (40.8)	16,019 (45.8)	15,344 (36.5)
0 or 1 favourable lifestyle behaviours	2477 (3.2)	1433 (4.1)	1044 (2.49)

PA, Physical activity, SD, standard deviation. ^1^ Townsend Deprivation Index is a composite measure of deprivation based on unemployment, non-car ownership, non-home ownership, and household overcrowding. ^2^ Medication use was restricted to lipid lowering or blood pressure. ^3^ Non-smoker was defined as never or past smoker; no overweight/obesity was BMI < 25 kg/m^2^; physically active was defined as >150 min activity; healthy diet was defined as above the median Healthy Diet Indicator score of 2.0; not sedentary was defined as ≤7 h TV watching and/or computer use; favourable sleep duration was defined as 7–9 h.

**Table 2:** Cox-proportional hazard models for CVD and all-cause mortality and CVD events according to a favourable, intermediate or unfavourable Lifestyle Score (LS) and polygenic risk score in UK Biobank participants.

	Overall (*n* = 76,958)				Males (*n* = 34,968)				Females (*n* = 41,990)			
	Cases	HR	95% CI	*p*-Value	Cases	HR	95% CI	*p*-Value	Cases	HR	95% CI	*p*-Value
All-cause mortality	2408				1415				993			
Favourable LS	1120	1.00			594	1.00			526	1.00		
Intermediate LS	1146	1.31	1.21, 1.43	<0.001	721	1.33	1.20, 1.49	<0.001	425	1.29	1.14, 1.47	<0.001
Unfavourable LS	142	2.06	1.73, 2.45	<0.001	100	2.17	1.76, 2.69	<0.001	42	1.85	1.35, 2.54	<0.001
Polygenic risk score		1.00	0.97, 1.05	0.64		1.02	0.97, 1.08	0.64		1.00	0.93, 1.05	0.74
CVD mortality	364				263				101			
Favourable LS	161	1.00			108	1.00			53	1.00		
Intermediate LS	177	1.35	1.09, 1.67	0.007	133	1.35	1.05, 1.75	0.020	44	1.32	0.88, 1.96	0.18
Unfavourable LS	26	2.48	1.64, 3.76	<0.001	22	2.66	1.68, 4.21	<0.001	4	1.79	0.65, 4.95	0.26
Polygenic risk score		1.11	1.00, 1.23	0.05		1.13	1.00, 1.28	0.045		1.04	0.86, 1.27	0.68
Myocardial Infarction	1140				822				318			
Favourable LS	509	1.00			355	1.00			154	1.00		
Intermediate LS	560	1.34	1.19, 1.52	<0.001	410	1.27	1.10, 1.46	0.001	150	1.54	1.23, 1.92	<0.001
Unfavourable LS	71	2.12	1.65, 2.72	<0.001	57	2.11	1.60, 2.80	<0.001	14	2.00	1.16, 3.47	0.013
Polygenic risk score		1.35	1.27, 1.43	<0.001		1.42	1.33, 1.52	<0.001		1.19	1.06, 1.32	0.003
Stroke	748				447				301			
Favourable LS	374	1.00			222	1.00			152	1.00		
Intermediate LS	335	1.15	1.00, 1.34	0.059	201	1.00	0.83, 1.21	0.99	134	1.42	1.13, 1.79	0.003
Unfavourable HLS	39	1.74	1.25, 2.43	0.001	24	1.43	0.94, 2.18	0.10	15	2.37	1.39, 4.03	0.002
Polygenic risk score		1.02	0.95, 1.10	0.52		0.99	0.90, 1.08	0.75		1.08	0.97, 1.21	0.18

CVD, cardiovascular disease; Lifestyle Score based on smoking status, BMI, diet quality, physical activity, sleep duration and sedentary time. Favourable lifestyle was 4 or more health behaviours; intermediate lifestyle was 2 or 3 health behaviours; unfavourable lifestyle was 0 or 1 health behaviours. Analyses were adjusted for age (time scale), sex (when not used to stratify), deprivation (categorical). PRS was also adjusted for the first 8 principal components of ancestry and genotyping batch. Models include the main effects of diet quality and PRS without interaction terms.

## Data Availability

The genetic and phenotypic UK Biobank dataset supporting the conclusions of this article is available on application to the UK Biobank. This research used the UK Biobank Resource under Application 34894.

## Supplementary Materials

The following are available online at https://www.mdpi.com/article/10.3390/nu13124283/s1, Table S1: STROBE Statement—Checklist of items that should be included in reports of cohort studies, Table S2: Components and scoring methods of the Healthy Diet Indicator (HDI), Table S3: Comparison of participant characteristics between the excluded and analytic sample, Table S4: Cox-proportional hazard ratios and 95% CI for risk of all-cause mortality, CVD mortality and CVD events according to a healthy lifestyle score (continuous) in participants from the UK Biobank, Figure S1: Flow diagram of participants in the UK Biobank.
