# Supplementary material for: Unhealthy Lifestyle, Genetics and Risk of Cardiovascular Disease and Mortality in 76,958 Individuals from the UK Biobank Cohort Study
Source: Nutrients. 2021 Nov 27;13(12):4283. doi: 10.3390/nu13124283 (PMC8707428; doi:10.3390/nu13124283)
Supplement: Supplementary file 1 [file nutrients-13-04283-s001.zip › nutrients-1458611-supplementary.pdf]

TITLE: Unhealthy lifestyle, genetic risk and risk of cardiovascular disease and mortality in the UK Biobank cohort study

AUTHORS: Katherine M. Livingstone[1*], Gavin Abbott[1], Joey Ward[2], Steven J. Bowe[3]

AFFILIATIONS
1, Deakin University, Institute for Physical Activity and Nutrition Research, School of Exercise and Nutrition Sciences, Geelong, VIC 3220, Australia, k.livingstone@deakin.edu.au, gavin.abbott@deakin.edu.au
2, Institute of Health and Wellbeing, University of Glasgow, Glasgow, UK, Joey.Ward@glasgow.ac.uk
3, Deakin Biostatistics Unit, Deakin University, Geelong, VIC 3220, Australia, s.bowe@deakin.edu.au

CORRESPONDING AUTHOR: Dr Katherine Mary Livingstone, Deakin University, Institute for Physical Activity and Nutrition, School of Exercise and Nutrition Sciences, Melbourne, Melbourne Burwood Campus, 221 Burwood Highway, Victoria 3125, Australia, Tel: +61 3 92445416 Email: k.livingstone@deakin.edu.au

Table S1. STROBE Statement—Checklist of items that should be included in reports of ***cohort studies***

	Item No	Recommendation	Page
**Title and abstract**	1	(*a*) Indicate the study's design with a commonly used term in the title or the abstract	1
		(*b*) Provide in the abstract an informative and balanced summary of what was done and what was found	
**Introduction**			
Background/rationale	2	Explain the scientific background and rationale for the investigation being reported	2
Objectives	3	State specific objectives, including any prespecified hypotheses	3
**Methods**			
Study design	4	Present key elements of study design early in the paper	3-4
Setting	5	Describe the setting, locations, and relevant dates, including periods of recruitment, exposure, follow-up, and data collection	3-4
Participants	6	(*a*) Give the eligibility criteria, and the sources and methods of selection of participants. Describe methods of follow-up	3-4
		(*b*) For matched studies, give matching criteria and number of exposed and unexposed	
Variables	7	Clearly define all outcomes, exposures, predictors, potential confounders, and effect modifiers. Give diagnostic criteria, if applicable	4-9
Data sources/ measurement	8*	For each variable of interest, give sources of data and details of methods of assessment (measurement). Describe comparability of assessment methods if there is more than one group	4-9
Bias	9	Describe any efforts to address potential sources of bias	9
Study size	10	Explain how the study size was arrived at	10
Quantitative variables	11	Explain how quantitative variables were handled in the analyses. If applicable, describe which groupings were chosen and why	9-10
Statistical methods	12	(*a*) Describe all statistical methods, including those used to control for confounding	9-10
		(*b*) Describe any methods used to examine subgroups and interactions	
		(*c*) Explain how missing data were addressed	
		(*d*) If applicable, explain how loss to follow-up was addressed	
		(*e*) Describe any sensitivity analyses	
**Results**			
Participants	13*	(a) Report numbers of individuals at each stage of study—eg numbers potentially eligible, examined for eligibility, confirmed eligible, included in the study, completing follow-up, and analysed	10

		(b) Give reasons for non-participation at each stage<br>(c) Consider use of a flow diagram	
Descriptive data	14*	(a) Give characteristics of study participants (eg demographic, clinical, social) and information on exposures and potential confounders<br>(b) Indicate number of participants with missing data for each variable of interest<br>(c) Summarise follow-up time (eg, average and total amount)	10-11
Outcome data	15*	Report numbers of outcome events or summary measures over time	11-13
Main results	16	(*a*) Give unadjusted estimates and, if applicable, confounder-adjusted estimates and their precision (eg, 95% confidence interval). Make clear which confounders were adjusted for and why they were included<br>(*b*) Report category boundaries when continuous variables were categorized<br>(*c*) If relevant, consider translating estimates of relative risk into absolute risk for a meaningful time period	11-13
Other analyses	17	Report other analyses done—eg analyses of subgroups and interactions, and sensitivity analyses	11-13
**Discussion**			
Key results	18	Summarise key results with reference to study objectives	13
Limitations	19	Discuss limitations of the study, taking into account sources of potential bias or imprecision. Discuss both direction and magnitude of any potential bias	14-15
Interpretation	20	Give a cautious overall interpretation of results considering objectives, limitations, multiplicity of analyses, results from similar studies, and other relevant evidence	14-15
Generalisability	21	Discuss the generalisability (external validity) of the study results	14-16
**Other information**			
Funding	22	Give the source of funding and the role of the funders for the present study and, if applicable, for the original study on which the present article is based	16

*Give information separately for exposed and unexposed groups.

Table S2. Components and scoring methods of the Healthy Diet Indicator (HDI)

Dietary Indictor	Indicator foods[1]	Criteria for scoring
1. Saturated fatty acids	Saturated fat	>10% energy intake=0<br>0-10% energy intake=1
2. Polyunsaturated fatty acids	Polyunsaturated fat	<6 or >10% energy intake=0<br>6-10% energy intake=1
3. Protein	Protein	<10 or >15% energy intake=0<br>10-15% energy intake=1
4. Total carbohydrates	Carbohydrates	<50% or >70% energy intake=0<br>50-70% energy intake=1
5. Dietary fibre	Englyst dietary fibre	<18 or >32 g/day=0<br>18-32 g/day =1
6. Fruits and vegetables	Mixed vegetable, vegetable pieces, avocado, beetroot, broccoli, butternut squash, cabbage/kale, carrot, cauliflower, celery, courgette, cucumber, garlic, leek, lettuce, mushroom, onion, olives, parsnip, pea, side salad, sweet pepper, spinach, sprouts, sweetcorn, fresh tomato, tinned tomato, green bean, turnip/swede, watercress, other vegetables, homemade soup (vegetables)<br>Stewed fruit, prune, dried fruit, mixed fruit, apple, banana, berry, cherry, grapefruit, grape, mango, melon, orange, satsuma, peach/nectarine intake, pear, pineapple, plum, other fruit	<400 g/day=0<br>≥400 g/day=1
7. Pulses and nuts	Baked bean, pulses, broad bean<br>Salted peanuts, unsalted peanuts, salted nuts, unsalted nuts, seeds, types of spreads/sauces consumed (peanut butter)	<30 g/day=0<br>≥30 g/day=1
8. Total non-milk extrinsic sugars	Total sugars	>10 % energy intake=0<br>0-10 % energy intake=1
9. Fish	Tinned tuna, oily fish, white fish, prawns, lobster/crab, shellfish, other fish<br>Homemade soup, ingredients in homemade soup (fish)	<32 g/day=0<br>≥32 g/day=1
10. Red meat and meat products	Beef, pork, lamb, other meat<br>Poultry intake (skin removed from poultry (no); fat removed from poultry(no))<br>Homemade soup, ingredients in homemade soup (meat)<br>Sausage, bacon, ham, liver	>90 g/day=0<br>≤90 g/day=1
11. Calcium	Calcium	<700 mg/day=0<br>≥700 mg/day=1

1. As available in the UK Biobank

Table S3. Comparison of participant characteristics between the excluded and analytic sample

Characteristic	Excluded<br>N (%)	Analytic<br>N (%)
N[1]	425,578	76,958
Sex, female	231,412 (54.4)	41,990 (54.6)
Age at recruitment (years), Mean ± SD	56.6 ± 8.2	56.2 ± 7.8
Townsend Deprivation Index		
Least deprived	82,549 (19.4)	18,115 (23.5)
2nd least deprived	82,883 (19.5)	17,222 (22.4)
Medium deprivation	84,328 (19.8)	16,062 (20.9)
2nd most deprived	85,484 (20.1)	14,891 (19.4)
Most deprived	89,711 (21.1)	10,668 (13.9)
Body Mass Index (kg/m^2), Mean ± SD	27.6 ± 4.9	26.5 ± 4.4
Favourable lifestyle behaviours2		
Non-smoker	374,612 (88.6)	71,995 (93.6)
No overweight/obesity	127,382 (29.9)	30,239 (39.3)
Physically active	206,533 (48.5)	23,131 (30.1)
Not sedentary	400,731 (94.2)	73,310 (95.3)
Optimal sleep	305,273 (72.3)	60,545 (78.7)

Townsend Deprivation Index is a composite measure of deprivation based on unemployment, non-car ownership, non-home ownership, and household overcrowding.
1, In the excluded sample, data on Townsend Deprivation Index, smoking and sleep were available in n=424,909, n= 422,628 and n=422,249, respectively.
2, Data for Healthy Diet Indicator are not presented as the variable was derived based on 2 or more OxforWebQs, which were not available in the excluded sample.

Table S4. Cox-proportional hazard ratios and 95% CI for risk of all-cause mortality, CVD mortality and CVD events according to a healthy lifestyle score (continuous) in participants from the UK Biobank

	Overall (n=76,958)				Males (n=34,968)				Females (n=41,990)			
	**Cases**	**HR**	**95% CI**	**P-value**	**Cases**	**HR**	**95% CI**	**P-value**	**Cases**	**HR**	**95% CI**	**P-value**
All-cause mortality	2,408	1.37	1.28, 1.46	<0.001	1,415	1.40	1.28, 1.53	<0.001	993	1.32	1.18, 1.47	<0.001
CVD mortality	364	1.45	1.22, 1.73	<0.001	263	1.50	1.22, 1.83	<0.001	101	1.32	0.94, 1.86	0.11
Myocardial Infarction	1,140	1.39	1.26, 1.54	<0.001	822	1.36	1.21, 1.52	<0.001	318	1.49	1.23, 1.79	<0.001
Stroke	748	1.22	1.08, 1.38	0.002	447	1.08	0.91, 1.26	0.14	301	1.47	1.21, 1.78	0.019

CVD, cardiovascular disease; Healthy Lifestyle Score was based on not smoking, no overweight/obesity, above median diet quality, meeting physical activity guidelines of 150 min/day, sleep duration between 7-9 hours and sedentary time ≤ 7 hours/day. Models were adjusted for age (time scale), sex (when not used to stratify) and deprivation (categorical).

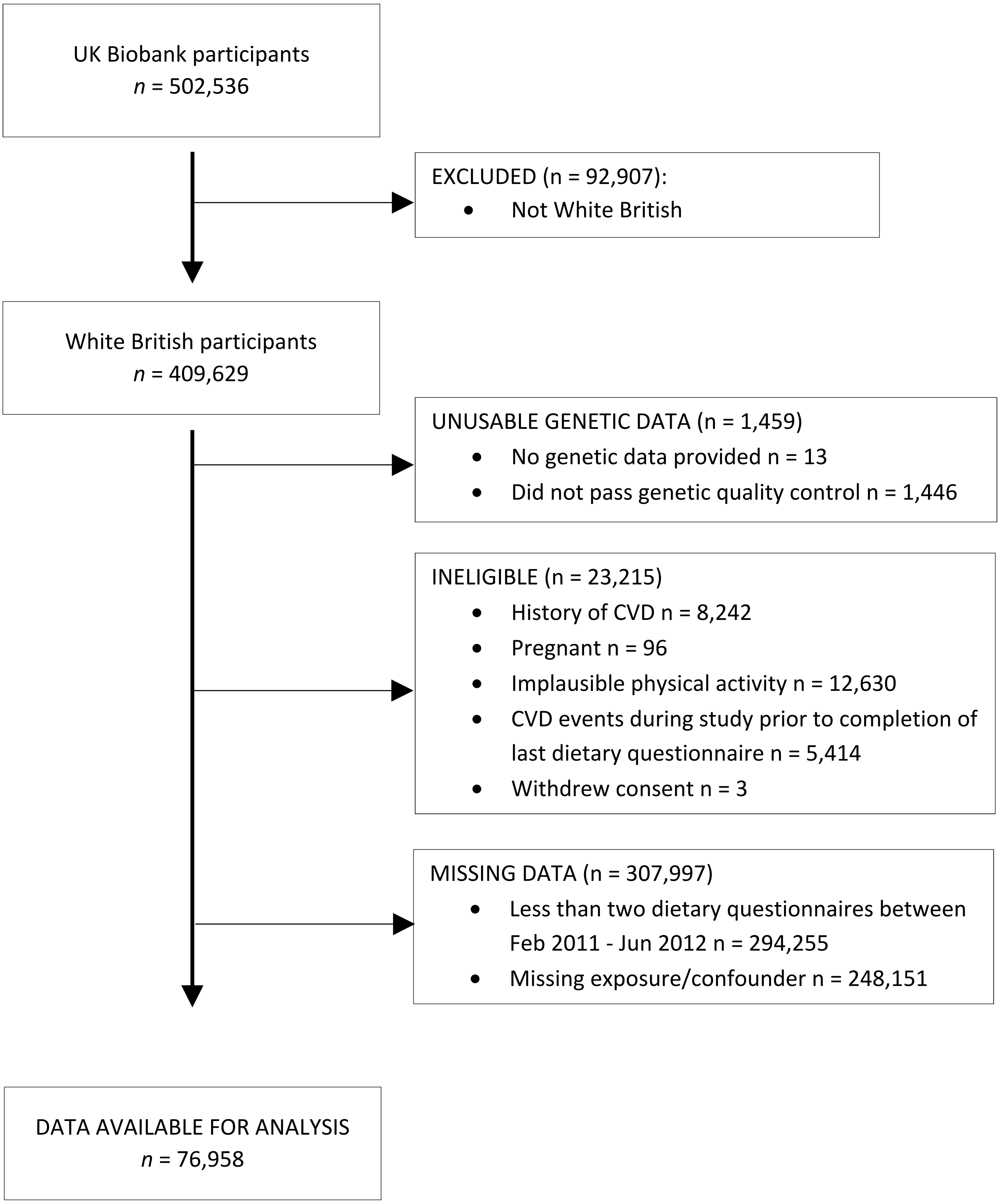

Figure S1. Flow diagram of participants in the UK Biobank
